# Schizophrenia genetics in the genome-wide era: a review of Japanese studies

**DOI:** 10.1038/s41537-017-0028-2

**Published:** 2017-08-30

**Authors:** Tetsufumi Kanazawa, Chad A. Bousman, Chenxing Liu, Ian P. Everall

**Affiliations:** 1 Department of Neuropsychiatry, Osaka Medical College, 2-7 Daigakumachi, Takatsuki, Osaka 569-8686 Japan; 2 Department of Psychiatry, University of Melbourne, Melbourne, 3052 VIC Australia; 3 Departments of Medical Genetics, Psychiatry, and Physiology & Pharmacology, University of Calgary, Calgary, AB Canada; 4 Institute of Psychiatry, Psychology and Neuroscience, King’s College London, DeCrespigny Park, London, SE5 8AF UK; 5 Department of Psychiatry, School of Medicine, Fujita Health University, Toyoake, Aichi 470-1192 Japan; 6 Department of Psychiatry, Shiga University of Medical Science, Otsu, Shiga 520-2121 Japan

## Abstract

The introduction of the genome-wide association study transformed schizophrenia genetics research and has promoted a genome-wide mindset that has stimulated the development of genomic technology, enabling departures from the traditional candidate gene approach. As result, we have witnessed a decade of major discoveries in schizophrenia genetics and the development of genome-wide approaches to the study of copy number variants. These genomic technologies have primarily been applied in populations of European descent. However, more recently both genome-wide association study and copy number variant studies in Asian populations have begun to emerge. In this invited review, we provide concise summaries of the schizophrenia genome-wide association study and copy number variant literature with specific focus on studies conducted in the Japanese population. When applicable, we compare findings observed in the Japanese population with those found in other populations. We conclude with recommendations for future research in schizophrenia genetics, relevant to Japan and beyond.

## Introduction

In 2007, a new era of schizophrenia research emerged with the publication of the first genome-wide association study (GWAS).^1^ The enthusiasm for the GWAS approach was immediately evident and it quickly gained traction within the field, with three schizophrenia GWASs published in 2008 (refs. 2–4) and another three in 2009.^5–7^ To date, greater than 30 schizophrenia GWASs have been conducted and most of these studies are now united within the Psychiatric Genomics Consortium. Collectively, these studies have independently implicated several genome regions in the pathophysiology of schizophrenia, most notably the major histocompatibility complex (MHC). The GWAS era has also spurred a genome-wide approach to the study of copy number variants (CNVs), which is beginning to give rise to our understanding of the impact of rare structural mutations on risk for schizophrenia. However, progress in the identification of genetic risk variants for schizophrenia, both common and rare, has been challenging, in part, due to inherent differences in genetic variation across different populations. As such, examination of ancestral homogeneous populations and/or rigorous control for population stratification in heterogeneous populations has become standard in the GWAS era, with results to date biased toward European populations. However, the last decade has witnessed a number of GWAS and CNV studies in non-European populations, particularly in Asian populations. Herein, we provide a concise review of the schizophrenia GWAS and CNV literature that has emerged over the past decade with specific focus on studies conducted in the Japanese population. When applicable, we compare findings observed in the Japanese population with those found in other populations. We conclude with recommendations for future research in schizophrenia genetics, relevant to Japan and beyond.

## Search strategy

We searched Google Scholar, MEDLINE, PubMed, and PsychINFO using the search terms schizophrenia, GWAS, CNV, genomics, gene, genetic, Japanese, and Japan with no language restrictions. Bibliographies of all research articles were hand-searched for additional references. All publications published from January 2007 through January 2017 were assessed for inclusion.

## GWAS in Japan

The first schizophrenia GWASs in the Japanese population were conducted in 2011 by Ikeda et al.^8^ and Yamada et al.^9^ (Table 1). The top hit in the Ikeda et al. study was rs11895771 in the *SULT6B1* gene, although it did not reach genome-wide significance (*p* = 8.0 × 10^−6^) and was not replicated in the validation sample (*p* = 0.14). However, meta-analysis of the Japanese samples with 479 cases and 2938 controls from a United Kingdom (UK) schizophrenia GWAS showed that the *SULT6B1* SNP (*p* = 3.7 × 10^−5^) as well as SNPs in the *GIRK2* (rs2787566, *p* = 0.0014) and *NOTCH4* (rs2071287, *p* = 0.0014) genes were associated with schizophrenia. The *NOTCH4* finding was subsequently supported in 2013 by Ikeda et al.^10^ in a large Japanese meta-analysis (6668 cases and 12,791 controls) that achieved genome-wide significance (*p* = 3.4 × 10^−8^). The *NOTCH4* gene is located within the MHC region (6p21.3–p22.1), a region repeatedly shown to harbor genetic risk variants for schizophrenia across populations.^11^ However, the portion of the MHC region (chr6: 28303247–28712247) implicated in schizophrenia risk by the Psychiatric Genomics Consortium GWAS^12^ does not include *NOTCH4*. Moreover, within the 128 SNPs identified by the Psychiatric Genomics Consortium GWAS,^12^ only 37 SNPs were genotyped (*n* = 8) or imputed (*n* = 14 by HapMap2, *n* = 15 by HapMap3) in the data set published by Ikeda et al. (575 cases and 564 controls)^8^ and none reached statistical significance (*p* < 0.0014 after Bonferroni correction; Supplementary Table 2), suggesting that schizophrenia risk loci are likely, in part, population-specific.

**Table 1:** Genome-wide studies in schizophrenia and related phenotypes within the Japanese population

Study type; phenotype; author (year)	Platform	Discovery sample	Validation sample	Top genes, Loci (*p* value)
Genome-wide association studies				
Schizophrenia				
Ikeda et al.^8^	Affymetrix 5.0	575 SCZ, 564 controls	1511 SCZ, 2451 controls	*SULT6B1*, rs11895771 (8.0 × 10^−6^)
Yamada et al.^9^	Affymetrix 100 K	120 Patient–parent trios	506 SCZ, 506 controls	*ELAVL2*, rs10491817 (8.7 × 10^−4^)
Cognition				
Hashimoto et al.^16^	Affymetrix 6.0	166 SCZ	—	*DEGS2*, rs7157599 (5.4 × 10^−7^)
Ohi et al.^17^	Affymetrix 6.0	411 Controls	257 SCZ	*TEK*, rs10757641 (3.62 × 10^−10^)
Atypical psychosis				
Kanazawa et al.^18^	Affymetrix 6.0	47 SCZ, 882 controls	560 SCZ cases, 548 controls, 107 BD cases, 107 controls	*CHN2/CPVL*, rs245914 (1.6 × 10^−7^)
Methamphetamine-induced psychosis				
Ikeda and Okahisa^10^	Affymetrix 5.0/6.0	194 METH-psychosis, 42 METH dependence, 864 controls	1108 SCZ subjects	*SGCZ*, rs4427170 (3.9 × 10^−6^)
Antipsychotic response/adverse event				
Ikeda et al.^21^	100 K SNP chip	99 first-episode SCZ	1564 SCZ, 3862 controls	*PDE7B*, rs9389370 (1.4 × 10^−3^)
Saito et al.^20^	Illumina HumanOmniExpress Exome v1.0/1.2	52 CIAG cases, 2948 controls	380 clozapine-tolerant subjects	*PBX2*, rs1800625 (3.46 × 10^−9^)
Copy number variant studies				
Schizophrenia				
Ikeda et al.^21^	Affymetrix 5.0	575 SCZ, 564 controls	—	Trend-level associations for 16p13.1, 1q21.1, and *NRXN1*
Kushima et al.^28^	NimbleGen 720 K	1699 SCZ, 824 controls	—	22q11.21 (2.6 × 10^−3^)

*BD* bipolar disorder, *CIAG* clozapine-induced agranulocytosis or granulocytopenia, *METH* methamphetamine, *SCZ* schizophrenia

Similar to the GWAS conducted by Ikeda et al.,^8^ Yamada et al.^9^ did not identify a SNP that reached genome-wide significance (*p* = 5.0 × 10^−8^). Using a three-stage approach the authors conducted a GWAS on 120 patient–parent trios and selected 1632 SNPs of nominal significance (*p* < 0.05). These selected SNPs were then examined in 506 cases and 506 age-matched and sex-matched controls from which the top SNP was rs10491817 in the *ELAVL2* gene (*p* = 8.7 × 10^−4^), a neuronal-specific RNA-binding protein involved in mRNA splicing and transcription regulation.^13^ The gene is known to bind to 3′ untranslated repeats and promote RNA degeneration.^14, 15^ They then conducted dense genotyping of the *ELAVL2* gene in 293 Chinese pedigrees (*n* = 1163) that showed a nominal association (lowest *p* = 0.026) in intron 1 with schizophrenia. Furthermore, *ELAVL2* was not identified in the most recent Psychiatric Genomics Consortium GWAS.^12^

### GWAS of associated phenotypes in schizophrenia

The GWAS approach in Japan has also been applied to a number of intermediate or broader phenotypes associated with schizophrenia, including cognitive functioning,^16, 17^ atypical psychosis,^18^ methamphetamine-induced psychosis,^19^ and severe antipsychotic adverse events^20^ and treatment response.^21^

Hashimoto et al.^16^ conducted a GWAS of cognitive decline among 166 Japanese individuals with schizophrenia. Cognitive decline was examined as a quantitative trait and calculated by subtracting premorbid IQ (Japanese Adult Reading Test) from current IQ (Wechsler Adult Intelligence Scale) for each participant. Genome-wide linear regression analysis identified rs7157599 (*p* = 5.4 × 10^−7^), a missense mutation in the *DEGS2* gene, as the strongest association with cognitive decline. While three additional SNPs (rs1555702, rs17069667, and rs1219705) were significant at a threshold of 5.0 × 10^−6^, one of them (rs17069667) is located in a GWAS-identified risk gene for schizophrenia (*CSMD1*).^12^ Building on this work, Ohi et al.^17^ conducted a GWAS of 52 cognitive phenotypes among 411 healthy controls and 257 individuals with schizophrenia. Among the healthy controls, the rs10757641 in the *TEK* gene had the strongest association (*p* = 3.62 × 10^−10^) with performance on the Visual Paired Associates II task, a measure of delayed memory. This association was not replicated in the schizophrenia sample, although gene-network analysis showed glutamate receptor activity False Discovery Rate (FDR; q = 4.49 × 10^−17^) and immune functions (FDR *q* = 8.76 × 10^−11^) were strongly associated with cognitive impairments in a broad range of domains among schizophrenia participants.

Specific psychosis phenotypes have also been examined. A GWAS approach was used to discover variants associated with “atypical psychosis^22^” or “Mitsuda psychosis” (similar to acute and transient psychosis, see Supplementary Table 1 for the diagnostic criterion) among 47 Japanese affected individuals compared to 882 healthy controls.^18^ The top-ranked SNP was rs245914 in the *CHN2* gene (*p* = 1.6 × 10^−7^), and several high-ranked SNPs in MHC region were detected. Further analysis of the SNPs associated with atypical psychosis suggested a significant enrichment for SNPs associated with schizophrenia but not bipolar disorder.

Another psychosis phenotype with a long history of candidate gene analyses in Japan is methamphetamine-induced psychosis.^23^ In 2013, the GWAS of methamphetamine-induced psychosis was conducted in Japan among methamphetamine-dependent individuals with (*n* = 194) and without psychosis (*n* = 42) along with 864 healthy controls.^19^ The strongest association with methamphetamine-induced psychosis was observed for rs12591257, an intronic SNP in the *AGBL1* gene (*p* = 3.6 × 10^−6^) that was identified as a risk gene for schizophrenia in the CATIE GWAS.^3^ In addition, polygenic component analysis revealed enrichment of schizophrenia risk alleles^8^ within the methamphetamine-induced psychosis sample (*p* = 0.009) but not in the sample of methamphetamine-dependent individuals without psychosis (*p* = 0.13).

Finally, the GWAS approach has been applied to the pharmacogenetics of antipsychotic efficacy and adverse events. In 2009, Ikeda et al.^21^ performed a convergent analysis using genome-wide pharmacogenetic and transcriptomic techniques to examine risperidone response (% the Positive and Negative Syndrome Scale (PANSS) change) among 108 first-episode schizophrenia patients. Results of both approaches identified 14 genes of potential relevance to risperidone response, among which a SNP (rs9389370) in the *PDE7B* gene, located in the MHC region, was replicated in three independent data sets comprising 1564 schizophrenia and 3862 normal controls (from Japan and UK). Interestingly, a recent small (*n* = 89) GWAS study in France^24^ also found that genetic variation in the MHC region was associated with psychotropic treatment response (% PANSS change) in schizophrenia, suggesting that the MHC region may harbor pharmacogenetic markers. In fact, the MHC region was further supported in a GWAS of clozapine-induced agranulocytosis or granulocytopenia (CIA/G; defined by the number of absolute neutrophil count). Saito et al.^20^ examined 50 Japanese CIA/G cases and 2905 healthy controls and found the strongest association in the *PBX2* (rs1800625, *p* = 3.5 × 10^−9^) and *NOTCH4* (three SNPs, *p* < 3.5 × 10^−8^) genes, both within the MHC region. Subsequent interrogation of this region by classical Human Leukocyte Antigen (HLA) typing showed greater prevalence of the HLA-B*59:01 allele in CIA/G compared to controls (*p* = 3.8 × 10^−8^, odds ratio (OR) = 10.7) and clozapine-tolerant schizophrenia patients (*p* = 3.0 × 10^−5^, OR = 6.3), although the authors noted that the effect was stronger for the CIA group (*n* = 22) compared to the CIG group (*n* = 28). Furthermore, their results suggested a trend-level association with the glutamate receptor gene, *GRM7* (rs3749448, *p* = 1.6 × 10^−6^).

In sum, the schizophrenia-related GWASs conducted to date within the Japanese population have examined a diverse range of phenotypes and have used small to moderate sized samples in their investigations. However, common threads of evidence have emerged from this work that implicate the MHC region (immune function) and the glutamatergic system as candidates for further exploration in schizophrenia and related phenotypes. In fact, the Japanese GWAS results, in particular those related to the MHC region and glutametergic system, are supported by the largest and most recent schizophrenia GWAS despite only 3.5% of this study comprising East Asians.^12^

## CNV studies in Japan

CNVs are a critical factor in the etiology of schizophrenia.^25, 26^ In Japan, two studies utilizing a genome-wide CNV approach have been conducted. The initial report led by Ikeda et al.^27^ analyzed 519 individuals with schizophrenia and 513 healthy controls and found no difference in global CNV burden between groups. However, in three regions (16p13.1, 1q21.1, and *NRXN1*) CNVs were more prominent among individuals with schizophrenia. In contrast, a subsequent study led by Kushima et al. that comprised 1699 cases and 824 controls^28^ reported clinically significant CNVs were threefold greater (OR = 3.04) in cases (9.0%) compared to controls (3.2%). These CNVs were enriched in pathways associated with oxidative stress response, genomic integrity, gene expression regulation, cell adhesion, neurotrophin signaling, kinase, synapse, small GTPase signaling, and endocytosis. In addition, nine of eleven previously reported gene sets associated with schizophrenia^11, 29–31^ were enriched for CNVs including four postsynapse-related, three presynapse-related, fragile X mental retardation protein targets, and calcium signaling. Of note, congenital abnormalities, such as heart defects, and/or premorbid developmental problems, such as intellectual disability were found in 42% of cases with CNV alternations.

These findings in the Japanese population have, in part, been replicated and extended in the most recent and largest European (21,094 cases and 20,227 controls) analysis of CNVs in schizophrenia.^32^ In this study, investigators identified a 11% greater global CNV burden in cases compared to controls (*p* = 5.7 × 10^−15^) among which eight loci, 1q21.1, 2p16.3 (NRXN1), 3q29, 7q11.2, 15q13.3, distal 16p11.2, proximal 16p11.2, and 22q11.2, obtained genome-wide significance. However, less than 1% (0.85%) of the variance in schizophrenia liability was explained by carrying a CNV in one of these eight loci and when combined with the schizophrenia liability explained by the 108 loci in the largest GWAS,^12^ the total proportion explained remains below 5%. Nevertheless, CNV and GWAS approaches remain important tools in the hunt for loci and genes associated with schizophrenia.

## Future directions

To guide future schizophrenia genetics research within and beyond Japanese populations and assist in prioritizing a strategy forward, we have generated the following recommendations.

### In-depth interrogation of the MHC region

**Fig. 1:**
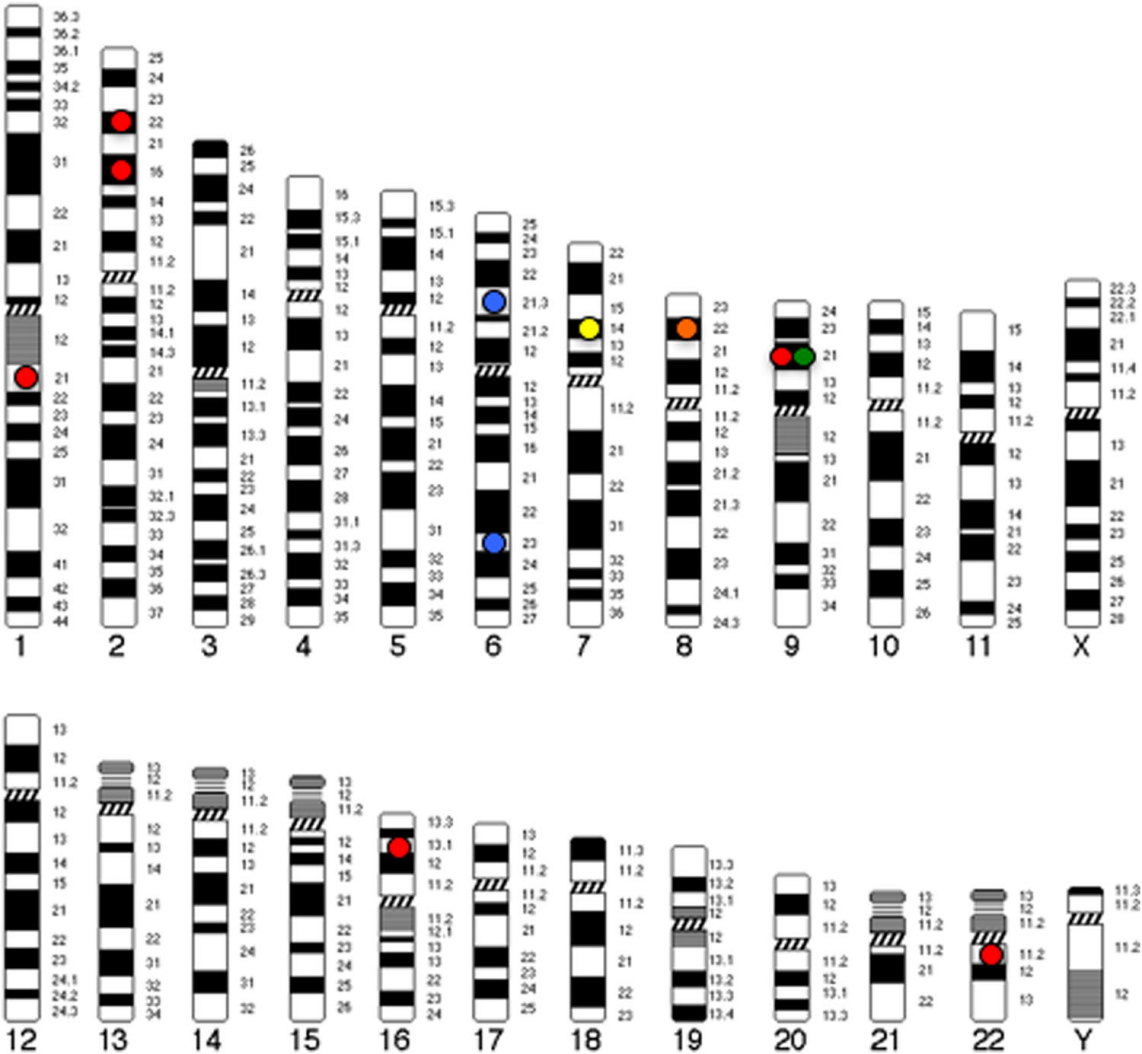
Genomic map of top loci identified in genome-wide studies of schizophrenia and related phenotypes within the Japanese population. *Circles* indicate candidate loci and *color* represents phenotype (Red circle=schizophrenia; Green circle=cognition; Yellow circle=atypical psychosis; Orange circle=methamphetamine-induced psychosis; Blue circle=antipsychotic response/adverse event). Genes (genomic regions) presented include: *SULT6B1* (2p22.2); *ELAVL2* (9p21.3); *DEGS2* (14q32.2); *TEK* (9p21.2); *CHN2/CPVL* (7p14.3); *SGCZ* (8p22); *PDE7B* (6q23.3); *PBX2* (6p21.32); *NRXN1* (2p16.3); 16p13.1; 1q21.1, 22q11.21

While a number of genomic regions have been implicated in schizophrenia and related phenotypes within the Japanese population (Fig. 1), the enrichment of risk variants within the MHC region (6p21.3–p22.1) is one of the most consistent findings in schizophrenia and is reported repeatedly in the Japanese studies we reviewed above. Within the NHGRI-EBI GWAS catalog,^33^ 341 SNPs have been recorded above the genome-wide significance (*p* < 5.0 × 10^−8^) for schizophrenia of which 12% (41 SNPs) and nine of the top 10 are located within the MHC region. This clearly signals the need for in-depth interrogation of this region in schizophrenia. As reported above, classical HLA typing has been undertaken in the context of clozapine-induced agranulocytosis in the Japanese population^20^ and others have identified excessive homozygosity in the MHC region of Ashkenazi Jews with schizophrenia, specially in a segment encompassed by *TRIM10, TRIM15*, and *TRIM40*.^34^ However, the mechanism by which these genes and more broadly the MHC region confer risk for schizophrenia remains largely undetermined. Although recent research suggests that the mechanism arises in part from many structurally diverse alleles of the complement component 4 (C4) gene,^35^ which is regulated by *CSMD1*, a gene with strong GWAS support. Yet, C4’s involvement in the etiology of schizophrenia within a Japanese population is unknown and warrants further investigation, particularly given known differences in allelic frequencies within the MHC region across populations.^36^ Further research along these lines is warranted, as the MHC region is complex and undoubtedly holds additional clues to the pathophysiology of schizophrenia.

### Identifying the missing heritability

The heritability of schizophrenia is widely cited to be ~80% but a recent GWAS estimated that common SNPs (minor allele frequency > 1%) alone explain ~23% of the variance in schizophrenia liability^37^ and CNVs likely contribute only modestly to this liability in the majority of individuals with schizophrenia.^38^ Thus, most of the heritability of schizophrenia remains unexplained, a phenomenon known as the missing heritability.^39, 40^

**Fig. 2:**
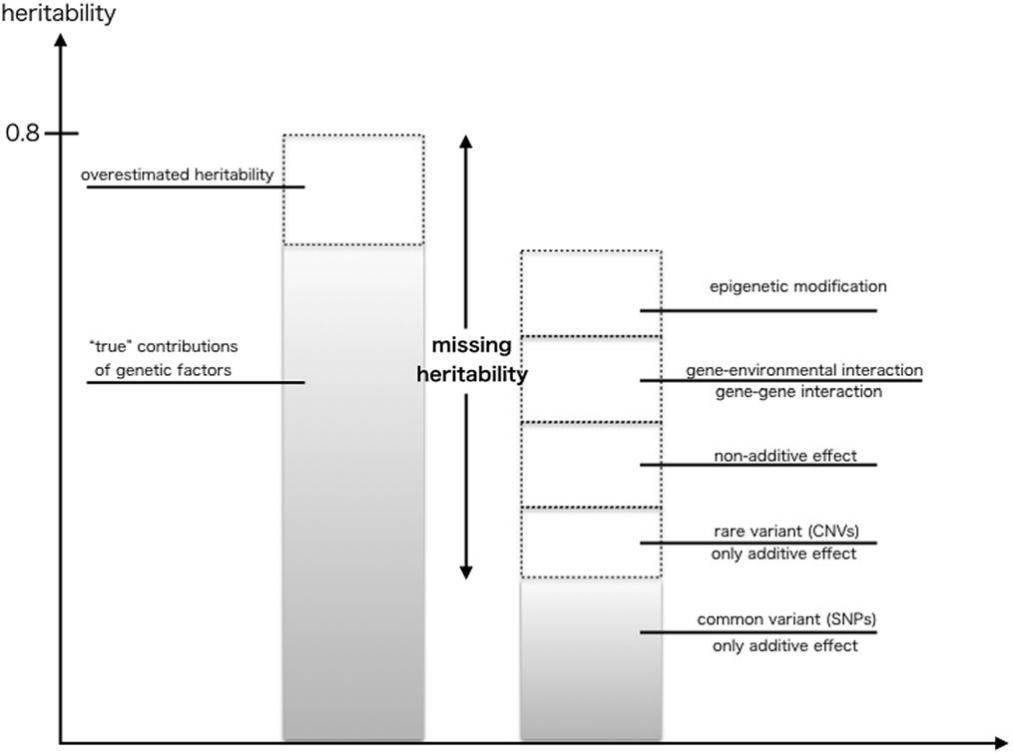
Possible mechanism of missing heritability. This figure shows the supposed mechanism of missing heritability. The percentage of each element will be varied across common disorders^44–46^

A number of strategies for identifying this missing heritability have been proposed over the past 5 years, including searches for rare and de novo variants using whole-genome approaches as well as whole epigenomic, gene–gene interaction, and gene–environment interaction studies (Fig. 2). However, studies of this nature require extremely large samples and for gene–environment interaction studies, rich environmental information, including in utero, infancy, childhood, and early adulthood exposures will be required. Growth of existing consortiums such as the European Network of National Networks studying Gene–Environment Interactions in Schizophrenia (EU-GEI^41^) will be crucial along with the development of methods for integrating and analyzing environmental and genomic data.

#### Looking beyond European populations

Schizophrenia genetic studies have predominately focused on people of European decent, despite a growing non-European population. In fact, people of Asian descent represent 3.5% (Japanese: 0.6%, Singapore: 1.2%, and Chinese: 1.7%) and 0% of the total samples included in the largest schizophrenia GWAS and CNV studies, respectively. Although genomic differences across populations are well documented, emerging evidence of clinically relevant genomic overlaps exist.^42^ Over the next decade more comparative research is required to identify both the common and unique genomic risk factors for schizophrenia, particularly as the world becomes more multicultural and clinicians will, if not already, begin caring for patients from a variety of genetic backgrounds. To facilitate this, large consortiums akin to the Psychiatric Genomics Consortium are needed for non-European populations. Such consortiums would provide a foundation for comparative genomic research in relation to schizophrenia and assist in determining the generalizability of current risk markers and polygenic risk scores beyond the European population. Furthermore, it is important to note that even within presumed homogenous populations genetic subgroups exist. For example, within the Japanese population two population clusters have been identified, Northern (Hondo) and Southern (Ryukyu), which have the greatest non-synonymous SNP frequency differences within the MHC region.^43^ Thus, within presumably homogenous populations greater knowledge of genomic overlaps and differences as they relate to schizophrenia liability will be critical to the success of efforts to translate genomic findings into the clinical setting.

## Summary

The genome-wide era has already delivered a significant number of biological insights into the pathophysiology of schizophrenia as well as other psychiatric disorders. In the European population we now have 108 GWAS loci as well as eight CNV loci for further interrogation. In the Japanese population, some of these loci (MHC region, *CSMD1*, *GRM7*) have been associated with schizophrenia but the majority of current candidate loci have yet to be fully characterized. As the number of studies in Japanese and other Asian populations increases, the sample sizes in current consortiums will reach a critical mass in which mega-analyses and/or meta-analyses akin to those recently completed among European populations will be possible. Until then, targeted replication studies within the Japanese and other Asian populations will aide in determining the generalizability of candidate GWAS and CNV loci for schizophrenia.

## Electronic supplementary material

Supplementary Table 1

Supplementary Table 2

## References

[1] Lencz T (2007). Converging evidence for a pseudoautosomal cytokine receptor gene locus in schizophrenia. Mol. Psychiatry.

[2] Shifman S (2008). Genome-wide association identifies a common variant in the reelin gene that increases the risk of schizophrenia only in women. PLoS Genet..

[3] Sullivan PF (2008). Genomewide association for schizophrenia in the CATIE study: results of stage 1. Mol. Psychiatry.

[4] O’Donovan MC (2008). Identification of loci associated with schizophrenia by genome-wide association and follow-up. Nat. Genet..

[5] Stefansson H (2009). Common variants conferring risk of schizophrenia. Nature.

[6] Shi J (2009). Common variants on chromosome 6p22.1 are associated with schizophrenia. Nature.

[7] International Schizophrenia Consortium et al. Common polygenic variation contributes to risk of schizophrenia and bipolar disorder.* Nature* 460, 748–752 (2009). 10.1038/nature08185 PMC3912837 19571811

[8] Ikeda M (2011). Genome-wide association study of schizophrenia in a Japanese population. Biol. Psychiatry.

[9] Yamada K (2011). Genome-wide association study of schizophrenia in Japanese population. PLoS ONE.

[10] Ikeda M (2013). Genetic evidence for association between NOTCH4 and schizophrenia supported by a GWAS follow-up study in a Japanese population. Mol. Psychiatry.

[11] Schizophrenia Psychiatric Genome-Wide Association Study. Genome-wide association study identifies five new schizophrenia loci. *Nat. Genet.* **43**, 969–976 (2011). 10.1038/ng.940 PMC3303194 21926974

[12] Schizophrenia Working Group of the Psychiatric Genomics Consortium et al. Biological insights from 108 schizophrenia-associated genetic loci. *Nature* **511**, 421–427 (2014). 10.1038/nature13595 PMC4112379 25056061

[13] King PH, Levine TD, Fremeau RT, Keene JD (1994). Mammalian homologs of Drosophila ELAV localized to a neuronal subset can bind in vitro to the 3’ UTR of mRNA encoding the Id transcriptional repressor. J. Neurosci..

[14] Gao FB (1994). Selection of a subset of mRNAs from combinatorial 3’ untranslated region libraries using neuronal RNA-binding protein Hel-N1. Proc. Natl Acad. Sci. USA.

[15] Berto S (2016). ELAVL2-regulated transcriptional and splicing networks in human neurons link neurodevelopment and autism. Hum. Mol. Genet..

[16] Hashimoto R (2013). Genome-wide association study of cognitive decline in schizophrenia. Am. J. Psychiatry.

[17] Ohi K (2015). Glutamate networks implicate cognitive impairments in schizophrenia: genome-wide association studies of 52 cognitive phenotypes. Schizophr. Bull..

[18] Kanazawa T (2013). Genome-wide association study of atypical psychosis. American journal of medical genetics. Neuropsychiatr. Genet..

[19] Ikeda M (2013). Evidence for shared genetic risk between methamphetamine-induced psychosis and schizophrenia. Neuropsychopharmacology.

[20] Saito T (2016). Pharmacogenomic study of clozapine-induced agranulocytosis/granulocytopenia in a Japanese population. Biol. Psychiatry.

[21] Ikeda M (2010). Identification of novel candidate genes for treatment response to risperidone and susceptibility for schizophrenia: integrated analysis among pharmacogenomics, mouse expression, and genetic case-control association approaches. Biol. Psychiatry.

[22] Kawashige S (2008). An association study of the signal transducer and activator of transcription 6 gene with periodic psychosis. Psychiatr. Invest..

[23] Bousman CA, Glatt SJ, Everall IP, Tsuang MT (2009). Genetic association studies of methamphetamine use disorders: a systematic review and synthesis. Am. J. Med. Genet..

[24] Le Clerc S (2015). A double amino-acid change in the HLA-A peptide-binding groove is associated with response to psychotropic treatment in patients with schizophrenia. Transl. Psychiatry.

[25] Malhotra D, Sebat J (2012). CNVs: harbingers of a rare variant revolution in psychiatric genetics. Cell.

[26] Kirov G (2014). The penetrance of copy number variations for schizophrenia and developmental delay. Biol. Psychiatry.

[27] Ikeda M (2010). Copy number variation in schizophrenia in the Japanese population. Biol. Psychiatry.

[28] Kushima, I. et al. High-resolution copy number variation analysis of schizophrenia in Japan. *Mol. Psychiatry* doi:10.1038/mp.2016.88 (2016). 10.1038/mp.2016.88 27240532

[29] Kirov G (2012). De novo CNV analysis implicates specific abnormalities of postsynaptic signalling complexes in the pathogenesis of schizophrenia. Mol. Psychiatry.

[30] Szatkiewicz JP (2014). Copy number variation in schizophrenia in Sweden. Mol. Psychiatry.

[31] Darnell JC (2011). FMRP stalls ribosomal translocation on mRNAs linked to synaptic function and autism. Cell.

[32] Marshall CR, Howrigan DP, Merico D (2017). Contribution of copy number variants to schizophrenia from a genome-wide study of 41,321 subjects. Nat. Genet..

[33] Welter D (2009). The NHGRI GWAS Catalog, a curated resource of SNP-trait associations. Nucleic Acids Res..

[34] Mukherjee S (2014). Excess of homozygosity in the major histocompatibility complex in schizophrenia. Hum. Mol. Genet..

[35] Sekar A (2016). Schizophrenia risk from complex variation of complement component 4. Nature.

[36] González-Galarza FF (2015). Allele frequency net 2015 update: new features for HLA epitopes, KIR and disease and HLA adverse drug reaction associations. Nucleic Acids Res..

[37] Cross-Disorder Group of the Psychiatric Genomics Consortium, et al. (2013). Genetic relationship between five psychiatric disorders estimated from genome-wide SNPs. Nat. Genetics.

[38] Wellcome Trust Case Control, C. (2010). Genome-wide association study of CNVs in 16,000 cases of eight common diseases and 3,000 shared controls. Nature.

[39] Lee SH, Wray NR, Goddard ME, Visscher PM (2011). Estimating missing heritability for disease from genome-wide association studies. Am. J. Hum. Genet..

[40] Manolio TA (2009). Finding the missing heritability of complex diseases. Nature.

[41] European Network of National Networks studying Gene-Environment Interactions in Schizophrenia et al (2014). Identifying gene-environment interactions in schizophrenia: contemporary challenges for integrated, large-scale investigations. Schizophr. Bull..

[42] Liu, C. et al. Pathway-wide association study identifies five shared pathways associated with schizophrenia in three ancestral distinct populations. *Transl. Psychiatry* **7**, e1037 (2017). doi:10.1038/tp.2017.8 10.1038/tp.2017.8 PMC5438037 28221366

[43] Yamaguchi-Kabata Y (2008). Japanese population structure, based on SNP genotypes from 7003 individuals compared to other ethnic groups: effects on population-based association studies. Am. J. Hum. Genet..

[44] Hachiya T (2016). Roots of missing heritability and the possibilities of prediction of phenotype and risk for onset of disease. Exp. Med..

[45] Stringer, S., Nieman, D. H., Kahn, R. S., Derks, E. M. in *Genome-Wide Association Studies From Polymorphism to Personalized Medicine* (ed Krishnarao Appasani) (Cambridge University Press, 2016).

[46] Zuk O, Hechter E, Sunyaev SR, Lander ES (2012). The mystery of missing heritability: genetic interactions create phantom heritability. Proc. Natl Acad. Sci. USA.

