# Supplementary material for: Schizophrenia genetics in the genome-wide era: a review of Japanese studies
Source: NPJ Schizophr. 2017 Aug 30;3:27. doi: 10.1038/s41537-017-0028-2 (PMC5577232; doi:10.1038/s41537-017-0028-2)
Supplement: Supplementary file 1 — Supplementary Table 1 [file 41537_2017_28_MOESM1_ESM.docx]

**Supplementary Table 1.** Diagnostic criterion of “atypical psychosis” or “Mitsuda psychosis”

| A. | A sudden onset of psychotic symptoms( 2 weeks or less from a mentally healthy state to a clearly psychotic state that fulfills the criterion B) |  |
| --- | --- | --- |
| B. | Symptoms from at least two of the following categories, occurring simultaneously: | 1. Emotional Turmoil (a); |
|  |  | 2. Perplexity, and confusion of memory (b); |
|  |  | 3. Catatonic behaviour (c) or hallucinations or delusions. |
| C. | The total duration of the disorder does not exceed 3 months, and there is almost complete recovery to the premorbid level of functioning. |  |
|  | The diagnosis should be qualified as "provisional" until 3 months after the onset. |  |
| D. | The disturbance is not due to the direct physiological effects of a substance or a general medical condition. |  |

Annotations:

a. emotional turmoil: Characterized by intense feelings of happiness or ecstasy, overwhelming anxiety or marked irritability.

b. perplexity, and confusion of memory: Characterized by puzzlement over perceived disorganization of thought, misidentification of people or places, or incoherence of the train of thought, resulting in impairment in cognition of the environment and intellectual performance, leading to confused thought and behaviours.

c. catatonic behaviour: At least one of the following must be prominent:

(1) motoric immobility as evidenced by catalepsy or stupor

(2) excessive motor activity

(3) extreme negativism or mutism

(4) peculiarities of voluntary movement as evidenced by posturing, stereotyped movement, prominent mannerisms or prominent grimacing

(5) echolalia or echopraxia
